# The Community Engagement and Translational Research Speaker Series: An Innovative Model of Health Education

**DOI:** 10.4172/2161-0711.1000227

**Published:** 2013-07-24

**Authors:** Lori E Crosby, Teresa Smith, William D Parr, Monica J Mitchell

**Affiliations:** 1 Department of Pediatrics and Co-director of Training, Community Engagement Core, Center for Clinical and Translational Science and Training (CCTST), Cincinnati Children’s Hospital Medical Center, University of Cincinnati College of Medicine, Cincinnati, Ohio, USA; 2 Community Engagement Core, CCTST, Cincinnati Children’s Hospital Medical Center, University of Cincinnati, Cincinnati, Ohio, USA; 3 Department of Pediatrics and Co-director, Community Engagement Core, CCTST, Cincinnati Children’s Hospital Medical Center, University of Cincinnati College of Medicine, Cincinnati, Ohio, USA

**Keywords:** Health education, Bi-directional training, Community training, Training model, Community engagement

## Abstract

**Introduction:**

New models of health education are needed as research is becoming increasingly translational and as health care models are being applied to both medical and community settings. The Community Engagement and Translational Research Speaker Series is an innovative model for community health education that engages academic and community participants in shared learning.

**Method:**

Over the previous four years, eight Speaker Series events each consisting of three, distinct educational activities have been developed and implemented. Attendees provided ratings on each series event and a subset of them completed a knowledge and process evaluation.

**Results:**

The Speaker Series has been well attended by both academic and community representatives (N = 1,573). Evaluation data indicate that participants were highly satisfied across the three events (95%). Data also indicate that the Speaker Series met its intended goals of incorporating community feedback (91%) and increasing knowledge of community resources (98%), identifying health priorities (85%), and developing academic-community partnerships (95%).

**Conclusion:**

The Speaker Series has been evaluated positively by both academic and community representatives. This health education model is comprehensive and could be replicated by medical schools and universities striving to enhance community health education programs and curricula.

## Introduction

Health education within the Academic Health Center (AHC) has traditionally focused on educating physicians and medical professionals on clinical practice and research issues [1]. Emerging literature indicates that new models of health education are needed as research is becoming increasingly translational and as health care models are being applied to both medical and community settings [2]. Innovative health education strategies have been documented, including models that promote learning beyond the walls of the academic health centers, and programs that ensure academic and community engagement during training [3,4]. In this way, health professionals and community members can exchange dialogue on community health needs and how these needs can be addressed [5].

One of the goals of academic-community bi-directional training is to ensure that community members have input in developing the content and other aspects of health education to ensure relevance and inclusiveness. Integrated training provides opportunities for participants to expand their knowledge and collaborative networks [6]. Collaboration facilitates the transfer of existing knowledge as well as new knowledge that is generated as a result of the interaction [7,8]. Thus, effective trainings should engage participants with content that is relevant, presenters who are effective, and in the case of academic-community (bi-directional) trainings, they should engage both audiences in a way that fosters common understanding and collaboration [5]. Developing novel approaches to health education that ensure participants gain knowledge and skills that can be applied beyond the training environment is fundamental to advancing professional development and health outcomes [9]. Additionally, participants need to be aware of resources that can foster their development and ability to work collaboratively with others [10].

This paper describes an innovative model of academic-community health education that was developed as part of our Center for Clinical and Translational Science and Training’s (CCTST) Community Engagement Core. The Community Engagement and Translational Research Speaker Series have three components: an *Academic-Community Grand Rounds; Community Forum and Panel Discussion*; and *Community Keynote*. The goals of the Speaker Series are to: (1) provide community health education that engages academic and community audiences in shared learning; (2) provide relevant and effective training for participants in academic and community trainings; and (3) increase participants’ knowledge of community and research resources, community health priorities, and developing academic-community health partnerships.

The development of the Speaker Series is discussed. Data from academic and community participants are also presented.

## Materials and Methods

### Program development

The Community Engagement and Translational Research Speaker Series were developed in 2009 as a collaborative effort between the University of Cincinnati CCTST’s Community Engagement Core and its Community Partner Council (CPC). The CCTST Community Engagement Core consists of academic health center faculty and community representatives. The goal of the Community Engagement Core is to improve health outcomes and disparities through community research, training and partnerships. A vital part of the CCTST and its operation is its CPC, which is comprised of 32 academic and community members who serve in an advisory board capacity for the Community Engagement Core.

Through a series of meetings, the CPC and Community Engagement Core created a training model that engages academic and community audiences in shared learning experiences in both academic and community settings. The result is the Community Engagement and Translational Research Speaker Series; an innovative method for training academic faculty, staff and trainees together with community physicians and community representatives on topics relevant to community health, community engagement, translational science, health policy, practice and research. The three-part series includes: an *Academic-Community Grand Rounds*, the *Community Forum and Panel Discussion*, and the *Community Keynote*. The events present a common theme via different events (health education formats) which take place over a two day period.

A subcommittee with representatives from the CPC and Community Engagement Core plans and implements the Speaker Series through regular meetings (four to six times per year). Subcommittee members provide input on the topics, content, and format. The Speaker Series topics are selected based on community health needs, interests and participant feedback. The subcommittee identifies national speakers and assists with scheduling to reduce the potential for conflicts with other events. Finally, the subcommittee selects appropriate community venues to ensure inclusion and accessibility and reviews program evaluation data to ensure the success of future events. Table 1 summarizes the format and topics for each of the Speaker Series events.

### Educational activities

Each educational activity is distinct in focus and format; however, one of the things that make the Speaker Series unique is that each series features a national academic and community partnership. The purpose of the Academic-Community Grand Rounds is to provide education about ways to advance health through community-based participatory research, health policy, community health methods as well as ways to engage vulnerable populations in clinical research. The format is a two-hour session consisting of a mix of didactic instruction, experiential activities, case discussions, an interactive panel discussion, and opportunities for dialogue. The goals of the Community Forum and Panel Discussion are to educate community and academic participants about community health needs and effective strategies through dialogue and shared learning. The Community Forum begins with a round table featuring a panel of national and local experts followed by a Q & A session. Unlike the Community Forum, the purpose of the Community Keynote is to provide more in-depth education about a specific community health topic (e.g. health policy). The format is unique in that the Keynote is followed by a ceremony recognizing the accomplishments of local academic-community partnerships and model programs.

### Setting and schedule

While the Grand Rounds activities are scheduled on the University Medical Campus, the Community Forum and Community Keynote activities are scheduled at community venues. Grand Rounds are scheduled in technology–equipped (e.g. Power Point, Webinar capabilities) student lecture halls in the medical school. One of our CPC organizations (e.g. United Way) hosts the Community Forum. At times, this has limited the number of participants due to space constraints; however, the planning committee feels that fostering the partnerships with CPC organizations outweighs the benefits of having more attendees. Suitable for an evening event, the Community Keynote is scheduled at a more formal community venue theater (e.g. Freedom Center). Venues are chosen based on accessibility, parking convenience, and disability accommodations. Two Speaker Series events are scheduled each calendar year, in the fall and spring. Academic faculty, staff and students are recruited *via* email and posted flyers displaying each event’s title and learning objectives approximately two weeks prior to the Speaker Series. Community physicians and representatives are also recruited via email sent to community Listservs and CPC members. Additionally, flyers are distributed at community events and meetings and emails are sent to all past Speaker Series attendees. All participants are encouraged to preregister for events for planning and evaluation purposes.

### Evaluations

Participants are asked to sign-in at the beginning of each educational activity. The sign-in sheet allows us to track attendance over time. Participants provide their name, contact information, and representative organization. In an effort to better understand the background of participants, we categorized them as either academic or community based on the data provided on the sign-in sheet. All participants complete an evaluation at the end of an educational activity. Because Continuing Medical Education (CME) credit is available for the Grand Rounds activity, the program evaluation is more focused on skill application in clinical practice settings. Despite this, some evaluation questions were asked across events (e.g. satisfaction with the educational event). This paper will report data for those questions. With respect to satisfaction, participants were asked to evaluate the program using either a 5-point or 7-point Likert scale. Using linear transformation we converted the data to a common 7-point Likert scale (1 = fair to 7 = excellent). Participants were also asked to rate their perceptions of how well the training increased their knowledge of community and research training resources, community health priorities, and ways to develop academic-community health partnerships using a 5-point Likert scale (1=poor to 5=excellent). A subset of Speaker Series attendees (n = 54) were asked to complete an evaluation that asked them about their knowledge of community and research resources, community health priorities, and strategies for developing academic-community health partnerships. To ensure that the Speaker Series remains relevant to community needs and incorporates community feedback, we asked as subset (n = 54) of CPC and Community Engagement Core members to complete a process evaluation. We used descriptive analyses to analyze the program evaluation data. Please note that our program evaluations are exempt from the University Institutional Review Board’s approval because they have been deemed performance improvement.

## Results

### Engages academic and community audiences in shared learning

**Figure 1:**
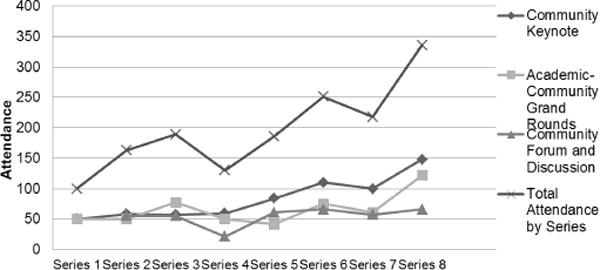
Speaker Series attendance totals.

A total of 1,573 academic and community representatives have attended the eight Speaker Series events (Figure 1). The overall attendance for each series increased over time, as did attendance for each of the three events. In addition, the Speaker Series was successful in integrating academic members in community events and community members in academic events, creating additional opportunities for shared learning and networking.

### Relevant and effective education

**Figure 2:**
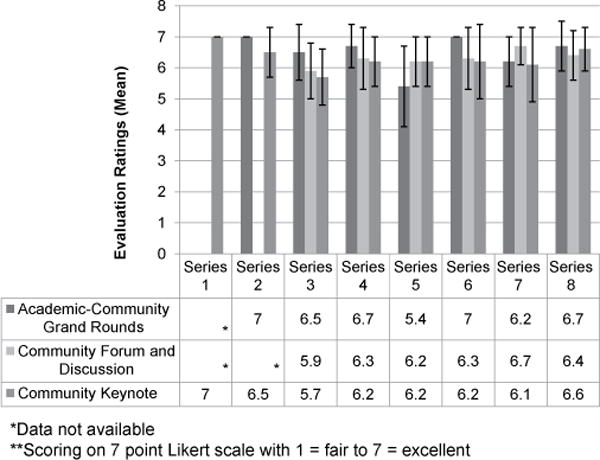
Overall evaluation of Speaker Series programs.

The percentage of academic participants attending the Community Forum and Community Keynote has grown over the years with approximately 50% of the audience representing an academic institution (Table 2). Data from the program evaluations indicated that participants were highly satisfied with the overall Speaker Series events as participants rated the events as a 5.7 or higher on a 7 point Likert scale with 7 being excellent (Figure 2). Additional outcomes from year 4 of the Speaker Series found that 107 (93%) participants agreed that they gained new skills from the trainings and 109 (95%) agreed that they will be able to apply the skills gained. Also, 112 (97%) participants strongly agreed that the activity was relevant to them professionally or personally, and 113 (98%) agreed that the program would positively impact them. A subset of CPC and Community Engagement Core participants surveyed (91%; N=54) reported that the Speaker Series was meeting its intended objectives of involving community participants in planning and incorporating community feedback.

### Knowledge of community health resources, priorities and partnerships

Almost all participants (98%) reported that they learned about community training and research resources. In addition, 85% of participants reported a better understanding of health priorities, and 95% reported an increased understanding of strategies for developing academic-community partnerships.

## Discussion

Using a collaborative framework and process, the Community Engagement and Translational Research Speaker Series was developed to broaden the traditional model of academic health education. The model has been implemented and sustained while successfully engaging both academic and community members in education, interactive learning, and networking opportunities. Ensuring that academic health center foster bi-directional learning is an emerging best practice and including the input of community members is timely as factors such as content, location, timing and accommodations are rarely considered by institutional parties [11].

The literature supports that innovative and multi-disciplinary training is essential to advancing health and translating clinical practice and research to community settings [12]. In this way, professionals within academic health centers and community organizations can develop a common understanding and the collaboration needed to address complex health challenges. The Speaker Series developed by our CCTST and CPC provides a model for how academic health centers can consider broadening educational formats while ensuring that health education is inclusive and relevant to community health which is particularly important given the high rates of documented health disparities in underserved populations.

It should be noted that the Speaker Series was developed and refined through planning between the CCTST and its community partners as well as through continuous improvement and learning, especially at the onset of the initiative. During the launch of the Speaker Series (Series 1 and 2), we collected limited data which was utilized to build the framework for the Speaker Series. While Series 1 included community events, there was limited interaction between community and academic audiences. However, Series 1 focused on foundational aspects of community-engaged and translational research which became an underlying theme throughout subsequent Series. In Series 2, community members were more fully integrated into events serving as panelists and facilitators which served as the basis for the current model.

Although preliminary data support its effectiveness, the Speaker Series has a number of limitations. First, more detailed data was collected in year 4 compared to years 1 through 3 making sample sizes smaller in those years. Second, we coded participants as either community or academic based on the organization they listed; however, in future evaluations, we will ask participants to self-identify as a community or academic member to strengthen reporting. Third, the cost of the Speaker Series can range from $3000 to $6000 depending on the speaker honoraria and venue which should be considered when replicating. Finally, we include a sub sample in the process evaluation which will be broadened in the future. Program evaluations will also include a broader representation of participations and will detail impact questions related to participation in community-academic health partnerships or translational research activities following participation in Speaker Series events. This would provide additional information about learners and guide future selection of topics. Finally, we have not evaluated how the Speaker Series may teach participants specific skills they can use to participate in community health research.

Despite these limitations, the Speaker Series has been sustained as the program continues to be implemented through the CCTST by the Community Partner Council. In addition, the Speaker Series has been evaluated positively by both academic and community participants. While many medical schools or universities may offer workshops or courses on community and translational research, the Speaker Series provides a longitudinal format for ongoing education and engagement around these issues. In essence the Speaker Series has created a learning community where academic and community participants can dialogue about strategies for improving health outcomes and developing health policy together. The strength of the Speaker Series is its flexibility; topics can be tailored to fit with local or national community health priorities. In conclusion, the Speaker Series is a comprehensive health education model that could be replicated by other medical schools or universities striving to enhance their community health education curricula.

## Figures and Tables

**Table 1:** Speaker Series topics.

Speaker Series Number	Academic-Community Grand Rounds	Community Forum and Discussion	Community Keynote
**Series 1**	Improving Health Outcomes through Community Engagement: A Long and Winding Road	Improving Health Outcomes through Community Engaged Research	Improving Health Outcomes through Community Engagement
**Series 2**	Community-Based Participatory Research: A Partnership Approach to Health Research and Intervention	Improving Health Through Partnerships and Community-Based Participatory Research	Eliminating Health Inequities through Community-Based Participatory Research
**Series 3**	Engaging and Retaining Community and Minority Populations in Clinical Research: Strategies and Opportunities	Engaging and Retaining Community and Minority Populations in Clinical Research: Strategies and Opportunities	Engaging Community and Minority Populations to Improve Health and Health Disparities
**Series 4**	The Why & How of Sustaining Community-Academic Health Research Partnerships	Creating Healthy Communities: Thinking and Working Together “Outside of the Box”	Transforming the Health of our Community Together
**Series 5**	Partnering with Communities to Improve Health through Translational Science	Building on Assets to Improve Health in our Community	Improving Health through Effective Community Partnerships: What We Know & What We Don’t Know
**Series 6**	Engaging Communities in Research Partnerships & Policy: Decreasing Health & Equity Disparities	Building Alliances to Improve Health & Transform Communities	The Power of Health Partnerships & Policy
**Series 7**	Community Assets, Resources & Partnerships: Leveraging Community Resources for Health	Innovative and Collaborative Models for Addressing Health Disparities in Our Community	Power in Communities to Improve Health
**Series 8**	A Comprehensive, Partnered Approach to Obesity Prevention: Lessons from Philadelphia	A Comprehensive, Partnered Approach to Obesity Prevention: Lessons from Philadelphia	Collaborative Approaches to Disease Prevention & Intervention: A Public Health Perspective

**Table 2:** Speaker Series attendance and participant affiliations.

Academic-Community Grand Rounds			
Speaker Series	Total Attended	% of Community Participants	% of Academic Participants
**Series 1**	50	Data not available	Data not available
**Series 2**	50	Data not available	Data not available
**Series 3**	77	0%	100%
**Series 4**	50	12%	88%
**Series 5**	41	7%	93%
**Series 6**	75	17%	83%
**Series 7**	61	30%	70%
**Series 8**	122	14%	86%
**Community Forum and Discussion**			
**Speaker Series**	**Total Attended**	% **of Community Participants**	% **of Academic Participants**
**Series 2**	55	Data not available	Data not available
**Series 3**	55	58%	42%
**Series 4**	21	57%	43%
**Series 5**	61	61%	39%
**Series 6**	66	35%	65%
**Series 7**	57	42%	58%
**Series 8**	66	47%	53%
**Community Keynote**			
**Speaker Series**	**Total Attended**	% **of Community Participants**	% **of Academic Participants**
**Series 1**	50	Data not available	Data not available
**Series 2**	58	47%	53%
**Series 3**	57	40%	60%
**Series 4**	59	58%	42%
**Series 5**	84	45%	55%
**Series 6**	110	46%	54%
**Series 7**	100	54%	46%
**Series 8**	148	49%	51%

## References

[1] Rothman AI, Sibbald G (2002). Evaluating medical grand rounds. J Contin Educ Health Prof.

[2] Bower EA, Girard DE, Wessel K, Becker TM, Choi D (2008). Barriers to innovation in continuing medical education. J Contin Educ Health Prof.

[3] Green EP, Borkan JM, Pross SH, Adler SR, Nothnagle M (2010). Encouraging scholarship: medical school programs to promote student inquiry beyond the traditional medical curriculum. Acad Med.

[4] Fisher JA (2003). Medical training in community medicine: a comprehensive, academic, service-based curriculum. J Community Health.

[5] Israel BA, Schulz AJ, Parker EA, Becker AB (1998). Review of community-based research: assessing partnership approaches to improve public health. Annu Rev Public Health.

[6] Hardy C, Phillips N, Lawrence TB (2003). Resources, knowledge and influence: The organizational effects of interorganizational collaboration. Journal of Management Studies.

[7] Gulati R, Nohria N, Zaheer A (2000). Strategic networks. Strategic Management Journal.

[8] Mowery DC, Oxley JE, Silverman BS (1996). Strategic Alliances and Interfirm Knowledge Transfer. Strategic Management Journal.

[9] Davis D, Evans M, Jadad A, Perrier L, Rath D (2003). The case for knowledge translation: shortening the journey from evidence to effect. BMJ.

[10] Foster-Fishman PG, Berkowitz SL, Lounsbury DW, Jacobson S, Allen NA (2001). Building collaborative capacity in community coalitions: a review and integrative framework. Am J Community Psychol.

[11] Mann KV (2011). Theoretical perspectives in medical education: past experience and future possibilities. Med Educ.

[12] Baquet CR (2012). A Model for Bidirectional Community-Academic Engagement (CAE): Overview of Partnered Research, Capacity Enhancement, Systems Transformation, and Public Trust in Research. J Health Care Poor Underserved.

